# Adjuvant Radiotherapy after Surgical Excision in Keloids

**DOI:** 10.3390/medicina57070730

**Published:** 2021-07-19

**Authors:** Jeong Won Lee, Ki Ho Seol

**Affiliations:** Department of Radiation Oncology, Daegu Catholic University School of Medicine, Daegu 42472, Korea; gardenlee@cu.ac.kr

**Keywords:** keloid, adjuvant radiotherapy, surgery

## Abstract

Keloids are a benign fibroproliferative disease with a high tendency of recurrence. Keloids cause functional impairment, disfigurement, pruritus, and low quality of life. Many therapeutic options have been used for keloids. However, the high recurrence rates have led to the use of adjuvant therapy after surgical keloid excision. There are different radiotherapy regimens available, and the advantages and disadvantages of each are still unclear. The aim of this review is to explain the appropriate radiotherapy regimen for keloids as well as discuss the recent reports on keloid management with radiotherapy. Adjuvant radiotherapy after surgical excision for keloids yields excellent local control with tolerable side effects. Hypofractionated radiotherapy with a BED of more than 28 Gy (α/β value of 10) after excision is recommended in the light of its biologic background.

## 1. Introduction

Keloids are caused by interruption of the normal repair process and excessive scar formation following a skin injury such as piercing, trauma, and surgery. Keloids occur in 5% to 15% of wound healing cases [1]. They are classified as a benign disease with inflammatory conditions, but not as a tumor despite their fibroproliferative tendency [2]. These abnormal conditions can cause not only esthetic problems, owing to the presence of red, raised scars, but also uncomfortable symptoms such as itching, pain, and scar contracture. Unfortunately, spontaneous regression of keloids is rarely observed [3]. Several treatment modalities like interferons, intralesional steroid injections, cryotherapy, laser therapy, silicone gel sheets, pressure therapy, surgical excision, and radiotherapy have been used in the management of the keloids. However, a high recurrence rate (over 50%) persists with these approaches [4,5]. Most authors have reported that the recurrence rate of keloids following surgical excision alone can be as high as 50% to 80% [6,7]. Furthermore, it has previously been reported that surgical excision alone results in high recurrence rates of up to 100% and is thus ineffective as a treatment option for keloids [8,9], because the surgery itself may result in inflammation [2]. After the surgical excision of keloids, additional treatment modalities are needed, and radiotherapy has been used as a part of this combination therapy [7,10,11]. The international panel of experts on scar management stated that postoperative radiotherapy combined with surgical excision is the most effective treatment for keloids, even serious keloid lesions [12]. There are different radiotherapy regimens available, and the advantages and disadvantages of each are still unclear. We thus focus on adjuvant radiotherapy after excision for keloids in this review.

## 2. The Mechanism of Adjuvant Radiotherapy

Radiotherapy has been used in keloid treatment with or without surgery [12]. The mechanism by which radiotherapy exerts positive effects in the management of keloids is uncertain [13]. Keloids are characterized by abnormal overexpression of growth factors, such as platelet-derived growth factor, fibroblast growth factor, transforming growth factor-α, transforming growth factor-β, and interleukin, which are released during the wound healing process after injury. Transforming growth factor-β is especially important for the development of keloids because of the association with fibroblast proliferation and formation of extracellular matrix components like collagen [5,9,14]. Its release from fibroblasts is hindered by radiation [15,16]; and thus, radiotherapy can prevent collagen synthesis and fibroblast proliferation, ultimately leading to inhibition of keloid formation [17,18,19].

According to Flickinger, radiotherapy reduces the extent of both normal and excessive wound healing by targeting rapidly growing fibroblasts, mesenchymal cells, and inflammatory cells [20,21]. In keloids, postoperative radiotherapy, which is administered to a more radiosensitive immature target after surgical excision, can lead to a better response than primary radiotherapy alone, which is administered to a mature target [22]. Several previous studies have reported good local control rates of 67% to 98% after postoperative radiotherapy for keloid treatment [23,24,25], and the recurrence rate of keloids following adjuvant radiotherapy after surgical excision (<10% to 20%) was also shown to be significantly lower than that following treatment with primary radiotherapy alone [26,27].

## 3. The Time Interval for Adjuvant Radiotherapy

The time interval between surgical excision and radiotherapy is important but still controversial. Some studies have found that the time interval between surgical excision and radiotherapy did not affect treatment outcomes [13,28,29,30,31,32]. Hintz mentioned that a longer interval after excision is suitable for keloid treatment due to the fact that the radiosensitization effect can be timed to act during the proliferative stage of the cell cycle, but suggested that a short interval after surgery was effective only in earlobe keloids [33]. In contrast, other authors have suggested favorable disease control benefits of approximately 10% to 23% when radiotherapy is administered within 24 h after surgery [34,35,36]. The efficacy of a short time interval (24 h between surgery and adjuvant radiotherapy) is based on the above-mentioned prevention of fibroblast proliferation [17,18,19]. Therefore, adjuvant radiotherapy is widely used and initiated within 24 h after surgical excision [21].

## 4. Radiation Technique

In the past, the following radiotherapy modalities were used and shown to have similar success rates; many of these are still available today: external beam radiotherapy using either low-voltage photons from orthovoltage units or high-voltage electron or photon beams from linear accelerators; and brachytherapy, which can be applied with either low-dose rate iridium-192 seeds or by using a high-dose rate iridium-192 source with an after-loading machine [17].

**Figure 1:**
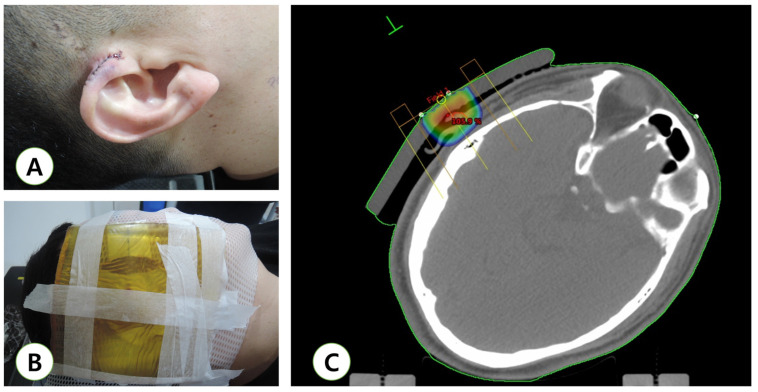
Example of computed tomography simulation for adjuvant radiotherapy using an electron beam. (**A**) Immediate post-operative view; (**B**) the patient was placed in a supine position and the head turned so that the ear to be treated was facing upward. A mask was used to immobilize the head. A part of the mask was excised to expose the area to be treated. A bolus was used to obtain a sufficient dose of the skin surface. The scar was marked with a wire. A margin of 1 cm around the scar was also marked with a wire to help with treatment planning; (**C**) computed tomography-based treatment plan for an ear keloid.

**Figure 2:**
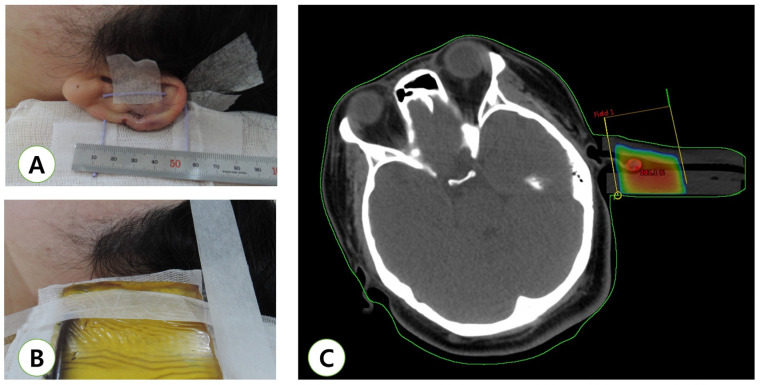
Example of adjuvant radiotherapy using a photon beam for an earlobe keloid. (**A**) Immediate post-operative view. (**B**) Simulation for radiotherapy. (**C**) Computed tomography-based treatment plan for an ear keloid.

Recently, several studies have supported the use of brachytherapy or electron irradiation [27,37,38,39,40]. However, these studies are limited by their retrospective designs, indirect comparison of radiation modalities, or old techniques that use kilovoltage X-rays. The type of radiation to be used, thus, remains controversial. In a recent meta-analysis, the following different types of radiotherapy were analyzed in only the adjuvant setting following excision: brachytherapy, electron therapy, and X-rays [27]. The results demonstrated that brachytherapy had superior local control outcome than X-rays (*p* = 0.04), but there was no statistically significant difference between brachytherapy and electron therapy (*p* = 0.10). Since it was not a direct comparative study, it cannot be concluded that brachytherapy is superior based on a single meta-analysis. Brachytherapy has the disadvantage of not having equipment in all hospitals and being invasive. Electron beam therapy is already widely utilized and recommended in the treatment of keloids [41]. However, the inhomogeneity of the electron beam irradiation dose distribution in areas with a large slope does not allow the treatment of concave or convex volumes with a homogeneous dose [42]. Depending on the location of the keloid, proper selection of electrons and photons is required to obtain an appropriate dose distribution. Thus, external beam radiotherapy using electrons (Figure 1) or photons (Figure 2) seems to be a good modality because of their easy availability, high feasibility, and less invasiveness, when compared with brachytherapy. Recently, new attempts such as the use of helical tomotherapy for complicated keloids to overcome the limitations of the conventional electron or photon therapy have also been reported [43].

The target volume usually includes a 10 mm radial margin around the scar and all the suture lines. The goal depth should be assessed using imaging devices prior to surgery (e.g., ultrasound), with an additional safety margin of at least 5–10 mm being provided [17]. Depending on the site of the scar and concern for irradiated dose to the adjacent tissue and organ, either a clinical or computed tomography simulation can be used. Depending on the expected dose distribution, bolus can be added to the irradiated area to provide an adequate surface dose to the scar. Lead shields or absorbers can be positioned near the field to block irradiated doses from reaching the adjacent critical structures. The actual selection of the planned radiotherapy technique is based on the location and size of the wound.

## 5. Radiation Dose and Fractionation

No research-based standardized treatment schedule exists for the use of post-surgical radiation despite radiation therapy having been in keloid treatment for decades. The optimal radiation dose and fractionation for keloids is still not known [2]. Kal and Veen hypothesized that an α/β value of 10 for keloids at the early responding tissues [18]. Use of the linear-quadratic model to calculate a biologically effective dose (BED) for various therapeutic adjuvant radiotherapy regimens showed that when a BED exceeds 30 Gy_10_, the recurrence rate is less than 10%. Therefore, they recommended a BED greater than 30 Gy_10_. On the other hand, Flickinger researched the α/β value of keloids and found it to be as low as two, thus demonstrating the efficacy of hypofractionated radiotherapy [20]. An in vitro study by Malaker et al. suggested a fraction size of 5 Gy for radiolysis of fibroblasts, but single irradiation with a large fraction size increases the possibility of treatment failure and skin necrosis [44,45]. Many authors have investigated the dose-dependent correlation of recurrence rate and have advocated 20 Gy in five fractions as an appropriate dose fractionation schedule [13,46]. Renz et al. reported the results of a retrospective study comparing the outcomes of two regimens of postoperative radiotherapy in 250 keloid lesions (125 keloids were treated with 20 Gy in five fractions while the other 125 keloids were treated with 12 to 16 Gy in 3 to 4 fractions) [46]. They reported that the lesions treated with a dose of 20 Gy had a recurrence rate of 1.6% compared with a rate of 9.6% in those lesions treated with <20 Gy (odds ratio: 0.16, *p* = 0.02). The BED of 20 Gy in five fractions was converted into 28 Gy_10_ or 60 Gy_2_ in a study by Flickinger [20]. Therefore, hypofractionated radiotherapy with a BED of more than 28 Gy (α/β value of 10) showed superior local control compared with treatment using lower dose regimens. Further investigation on fractionation regimen that effectively prevent recurrence without elevating the risk of secondary carcinogenesis is helpful.

## 6. Clinical Outcomes of Adjuvant Radiotherapy

**Figure 3:**
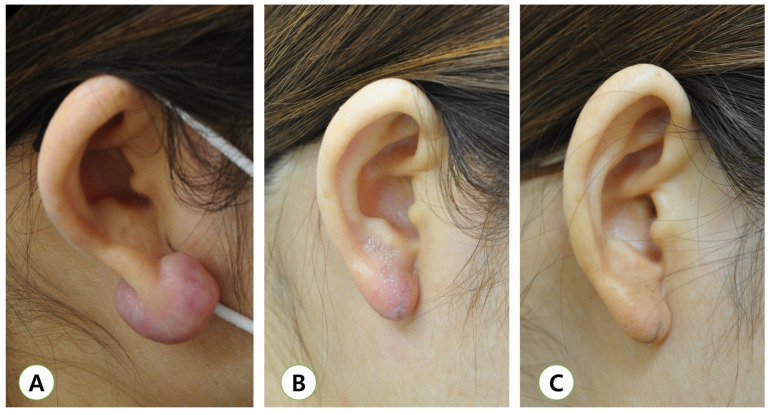
(**A**) Pre-operative view, (**B**) immediately post-radiotherapy (20 Gy in five fractions following excision of keloids). Radiotherapy was initiated within 24 h after the excision. No treatment-related complication was noted. (**C**) One-year follow-up image of following treatment with adjuvant radiotherapy. No recurrence of the elevation of the mass was noted.

Numerous studies have proved the contentable result of surgery followed by radiotherapy for the treatment of keloids. A literature review of over 70 studies displayed that radiotherapy after surgical excision significantly decreased recurrence rates (22% ± 4%) compared with radiotherapy alone (recurrence rate of 37% ± 12%) [27]. Figure 3 shows an example of successful earlobe keloid treatment with surgical excision and adjuvant radiotherapy.

The same meta-analysis revealed the different recurrence rates by the site of keloid lesions. In respect of the site of keloid lesion, the lesions located on the chest and trunk had the highest recurrence rate of 34% compared with the lesions on the ear, head and neck, or extremities. Keloids localized on the ear were found to have the lowest rate of recurrence (12%) [27]. The difference in the tensile strength of the skin in each of these areas may cause this variation in recurrence rates by the site of lesions.

## 7. Complications after Radiotherapy

In general, the application of relatively low total radiation doses does not stimulate skin and soft tissue reactions beyond the common toxicity criteria (CTC) level of Grade 1–2 [17]. Acute and late complications are generally minor, and the most common side effects are skin damage such as erythema, desquamation, and pigmentation. These were observed in 25% of cases reported in existing literature and were seen when higher total radiation dose was used [47,48]. An analysis of 194 patients with keloids reported that the incidence of moderate-to-severe skin reactions (hyperpigmentation, depigmentation, and telangiectasis) was 19%, but none of the patients had critical complications of CTC grade 3 or higher [13]. Other studies have demonstrated very low incidence rates of less than 1% or no incidence rate for serious complications with wound infection or dehiscence [27,34,49]. To improve and accelerate the skin regeneration process, the patient should be instructed to apply hydrating products, such as aloe vera gel or glycerol, locally over the lesion for a few weeks after the post-surgical dressing has been removed.

There is concern about the risk of secondary malignancies due to radiation exposure. After radiotherapy of keloid, the main risk to be considered is the induction of malignant disease by the radiation exposure of critical organs in the field and from scatter. Ogawa et al. found five cases recorded as radiation-induced malignant tumors in keloid patients [24]. However, the relationship with radiotherapy was ambiguous in most of them. Thus, they concluded that the risk of radiation-induced secondary malignancy is very slight. Other authors have stated no association between secondary malignancy and the use of radiotherapy in keloids [13,25,27,36,48]. Although the safety of the radiotherapy in keloid has been established, attention should be paid to radiosensitive organs such as thyroid gland and mammary gland in treatment planning and radiation delivery.

## 8. Conclusions

The present review summarizes the usefulness of adjuvant radiotherapy following surgical excision in the treatment of keloids. This overview will benefit practitioners by providing them evidence-based strategies for the treatment of keloids, that is, use of adjuvant radiotherapy following surgical excision.

Adjuvant radiotherapy after surgical excision for keloids yields excellent local control and has tolerable side effects. Hypofractionated radiotherapy with a BED of more than 28 Gy (α/β value of 10) within 24 h after excision is recommended in light of its biologic background.

## Data Availability

Not applicable.
